# Organic Cathodes, a Path toward Future Sustainable Batteries: Mirage or Realistic Future?

**DOI:** 10.1021/acs.chemmater.3c02408

**Published:** 2024-01-16

**Authors:** Jan Bitenc, Klemen Pirnat, Olivera Lužanin, Robert Dominko

**Affiliations:** † National Institute of Chemistry, Hajdrihova 19, 1000 Ljubljana, Slovenia; ‡ Faculty of Chemistry and Chemical Technology, University of Ljubljana, Večna pot 113, 1000 Ljubljana, Slovenia; § Alistore-European Research Institute, CNRS FR 3104, Hub de l’Energie, Rue Baudelocque, 80039 Amiens, France

## Abstract

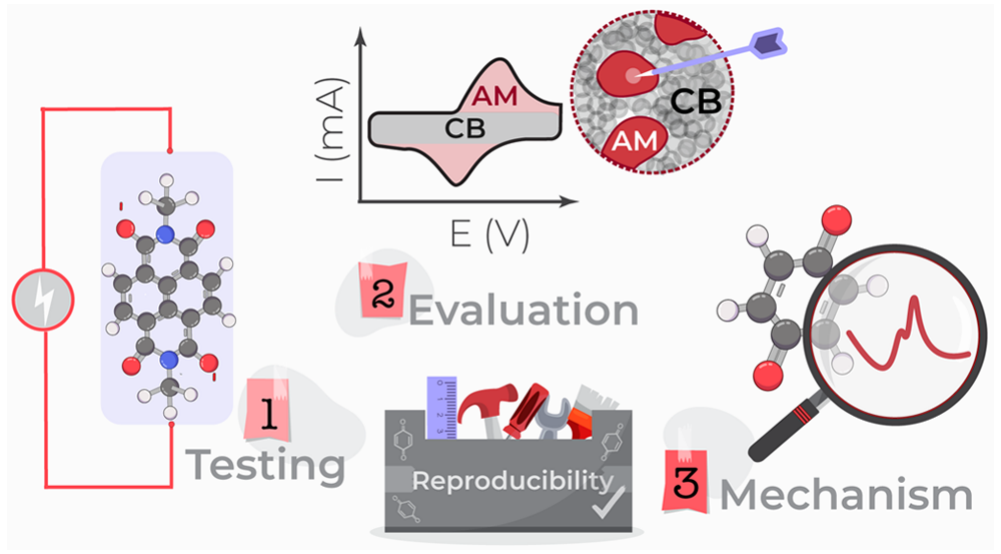

Organic active materials are seen as next-generation battery materials that could circumvent the sustainability and cost limitations connected with the current Li-ion battery technology while at the same time enabling novel battery functionalities like a bioderived feedstock, biodegradability, and mechanical flexibility. Many promising research results have recently been published. However, the reproducibility and comparison of the literature results are somehow limited due to highly variable electrode formulations and electrochemical testing conditions. In this Perspective, we provide a critical view of the organic cathode active materials and suggest future guidelines for electrochemical characterization, capacity evaluation, and mechanistic investigation to facilitate reproducibility and benchmarking of literature results, leading to the accelerated development of organic electrode active materials for practical applications.

## Introduction

Electrification of transportation has increased the demand for Li-ion batteries (LIBs), which, in turn, has led to many of the raw materials becoming listed as critical raw materials (CRMs). Currently, the European Union (EU) recognizes graphite, silicon, cobalt, and lithium as CRMs.^1^ The list of EU CRMs is likely to expand with the addition of nickel, which is already listed as a CRM by the U.S. Geological Survey.^2^ Hence, there is a pressing need for the development of high-energy density alternatives that would be based on abundant, sustainable, and cost-effective materials. The closest alternative to LIBs is a sodium-ion rocking chair battery, which can also act as a drop-in technology for current LIBs but with significantly decreased energy density.^3^ Other possible alternatives are alkali metal and multivalent metal anode batteries. The latter typically suffer from a lack of suitable cathode materials due to the inability to accommodate cations with a high charge density within the inorganic crystal lattice. In most cases, the structure is irreversibly transformed or converted into several phases. As an alternative, organic electrode materials allow us to circumvent the limitations encountered inside inorganic hosts and offer reversible electrochemical activity with ions of different sizes and charges.^4^ Operation with various metal ions opens up the possibility for extensive exploration of post-Li batteries based on more abundant metals (Na, K, Mg, Ca, and Al). Moreover, organic materials could potentially be produced from a biomass-derived feedstock and are typically synthesized at lower temperatures, contributing to an overall decrease in the CO_2_ footprint and more sustainable battery production.^5^ However, the production footprint might vary significantly from compound to compound depending on the synthesis route, the number of synthesis steps, and the utilized reagents.

**Figure 1:**
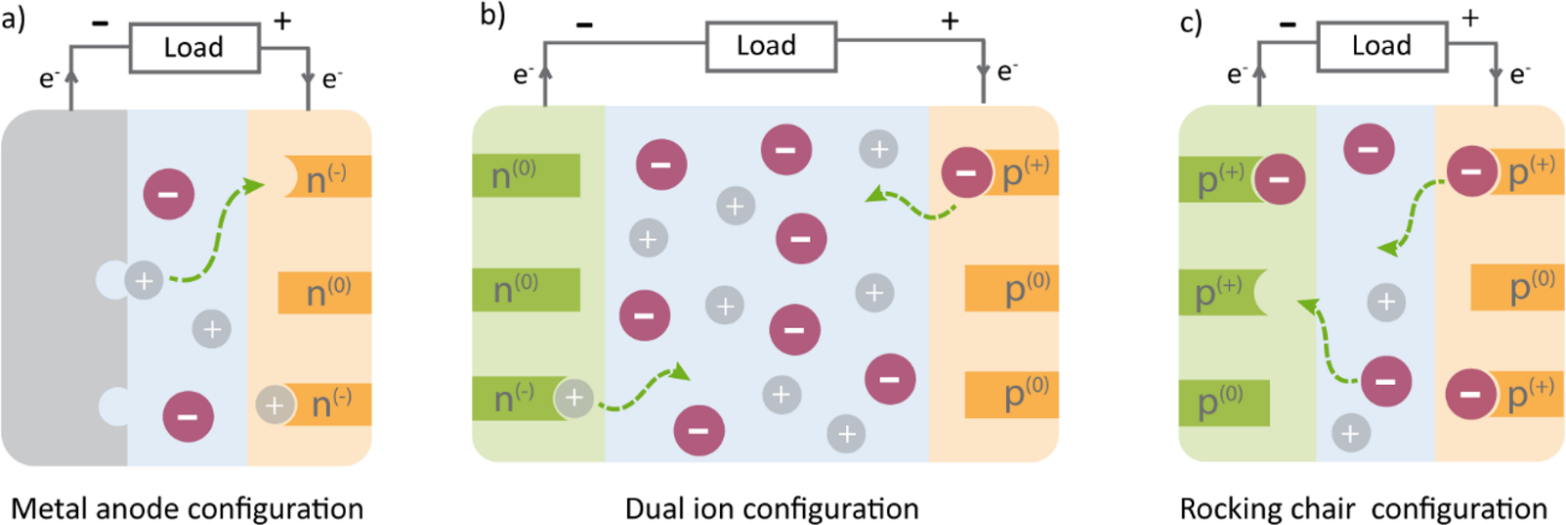
Schematic representation of different battery cell configurations employing organic electrode materials. (a) Configuration with a metal anode and an n-type organic cathode in which the electrolyte concentration remains constant. (b) Dual-ion configuration employing an n-type anode and a p-type cathode in which the electrolyte concentration increases in discharge and decreases in charge. (c) Rocking chair mechanism with two p-type electrodes in which electrolyte concentration remains constant. This figure was inspired by ref (6).

Organic electrode materials encompass a highly variable group of materials and can be classified in several ways. According to the electrochemical mechanism, they are classified into n-type, p-type, and bipolar (can act as both n- and p-type) (Figure 1). The specific electrochemical mechanism has an important influence on the type of cell that we can build. If we want to enable cation storage in combination with a metal anode under lean electrolyte conditions, n-type materials are needed as electrodes. Another option is dual-ion operation with mixed n- and p-type electrodes. However, dual-ion storage requires a large volume of the electrolyte to compensate for the charge accumulated on the electrodes during battery operation, which severely limits the practical energy density. P-Type materials can also be operated under lean electrolyte conditions but require the combination of a p-type anode and a cathode for the conventional rocking chair configuration. The primary focus of this Perspective will be the application of n-type compounds and their electrochemical performance with various cations for targeted application in combination with metal anodes (Li, Na, K, Mg, Al, Ca, etc.) enabling high energy densities, while other types of materials and cell configurations will be briefly mentioned.

The research on the application of organic materials in batteries was initiated in the 1980s. At the time, the research was mainly focused on the use of p-type conducting polymers and their application as cathodes in dual-ion configurations, with the organic polymer serving as a cathode.^6^ A more widespread application of organics was initiated at the beginning of the century with initial efforts on p-type polymers incorporating radical electroactive groups enabling high cycling rates,^7^ followed by research on high-capacity organic materials. Organic battery research and the development of organic materials have been comprehensively summarized in several recent reviews.^5,8−15^ It is not our aim to reiterate these efforts; instead, we aim to focus on some less often discussed aspects of the research on organic electrode materials.

In this contribution, we address the critical points in the research of organic electrodes. First, we discuss the role of organic electrode materials in the battery landscape and point out the need for the rigorous determination of the capacity of organic materials. This is followed by the evaluation of the obtained electrochemical capacity and the methods used to investigate the electrochemical mechanism. Toward the end, we discuss the post-Li metal–organic batteries. In this Perspective, we will not discuss the application of organic materials in redox flow batteries, as the requirements for these types of batteries differ significantly from those of conventional organic batteries. However, most of the points raised regarding electrochemical testing and electrochemical mechanistic investigation discussed on the topic of metal–organic batteries are valid for all organic materials and can be extended to other areas of research, including active materials for redox flow batteries. We hope this Perspective can provide useful guidance to academia and R&D, contributing to the accelerated development of organic materials through improved reproducibility and comparability of reported results.

## Role of Organic Electrode Materials in the Battery Landscape

Organic electrodes can be envisioned to play two very different roles. First, as a complementary technology that would be used to serve specific applications like portable electronics, robotics, or sensors where certain highly specific battery properties would be required (flexibility, sustainability, biodegradability, etc.). The second possible role is an alternative to ubiquitous and currently market-dominating LIBs in applications where volumetric energy density is not prioritized. The technological advancement of LIB technology has been achieved through the gradual progress in material chemistry, the use of various additives, electrodes, and cell engineering in the past three decades, which can also be, to a certain degree, envisioned for alternative battery technologies. Currently, the NMC 811 cathode material delivers an energy density of 760 Wh kg^–1^ (Table 1) on the level of the active cathode material (considering 3.8 V vs Li^+^/Li). To achieve a comparable gravimetric energy density, organic materials with high voltage or high specific capacity should be targeted. Ideally, the best candidate would possess both. At the same time, organic materials should deliver long-term stability comparable to that of conventional inorganic materials. Nevertheless, because of the low TRL and the primary emphasis on attaining optimal energy density, a noticeable lack of systematic work exists regarding prolonged durability, self-discharge, and aging of organic materials.

**Table 1:**
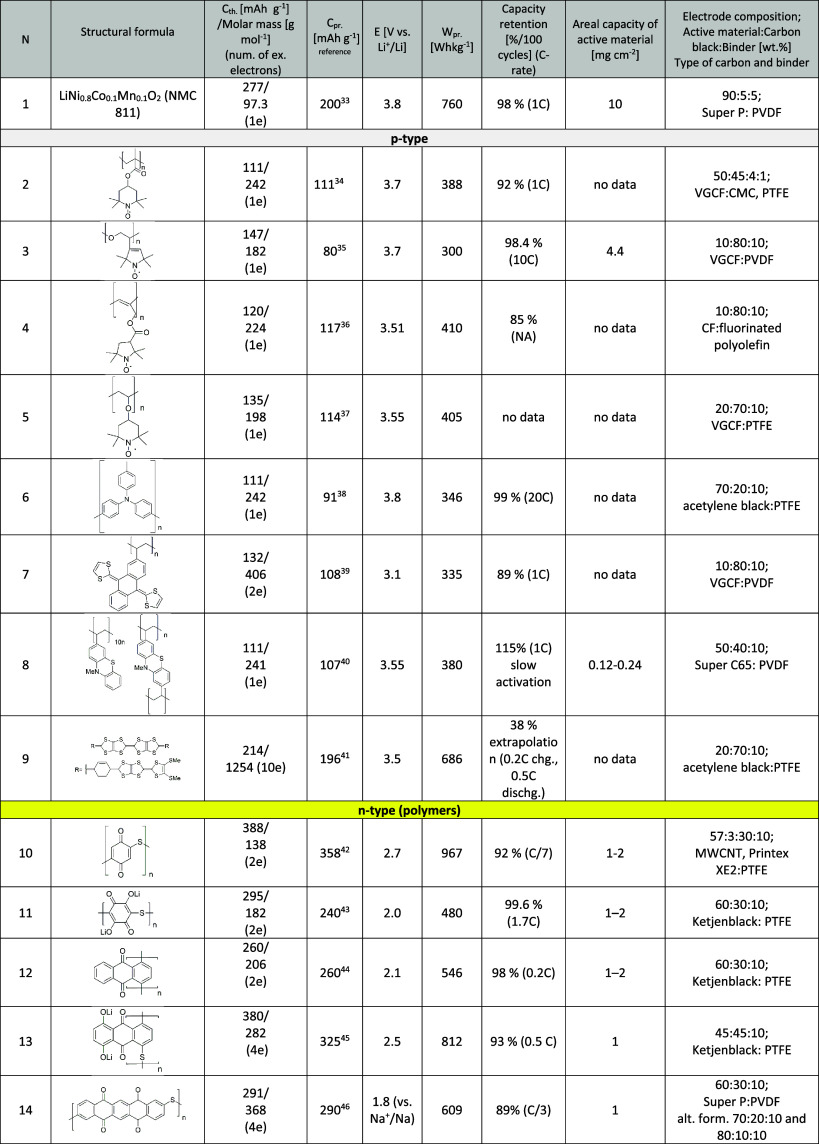

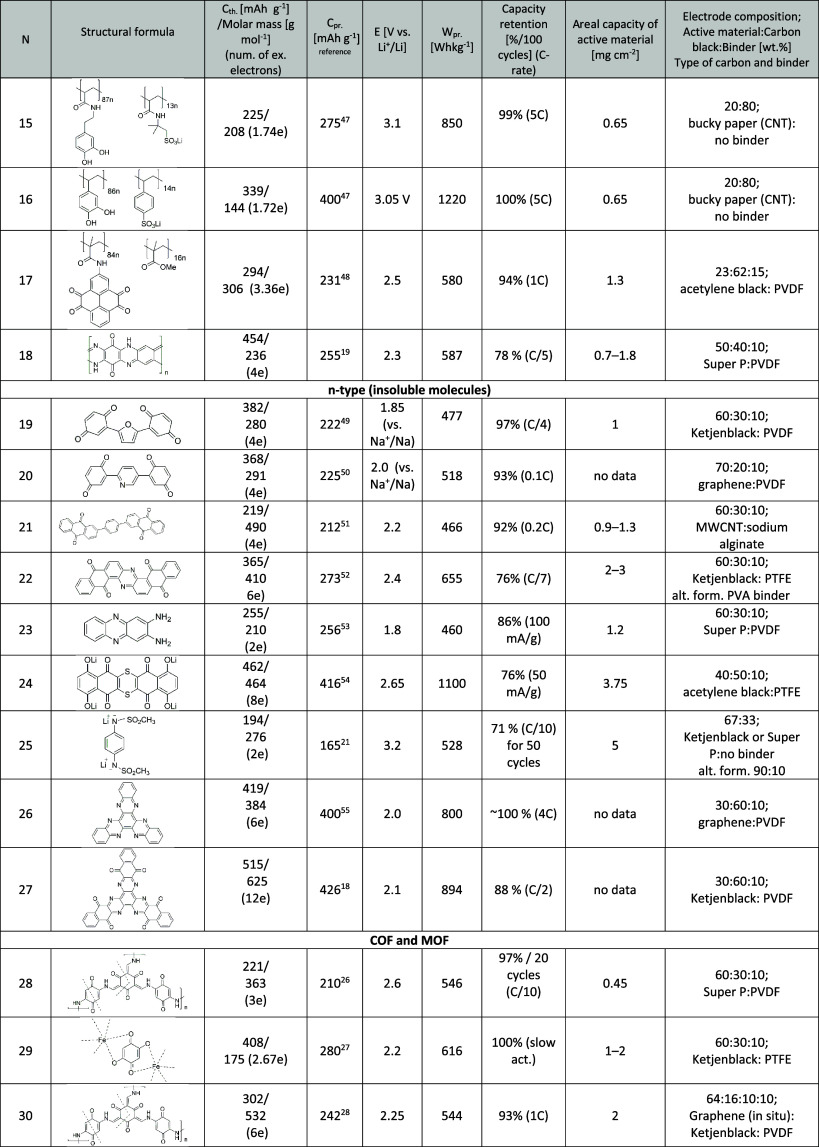

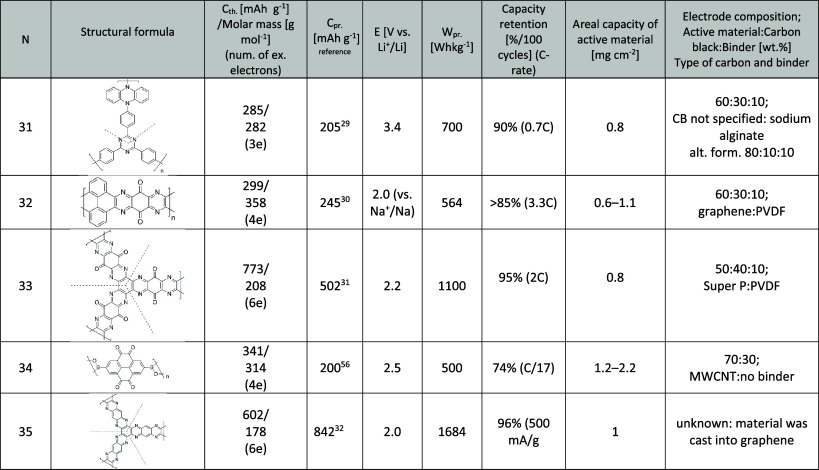
An Overview of the Representative Compounds from the Class of Organic Active Materials with Listed Theoretical and Practical Capacities, Redox Potentials, Gravimetric Energy Densities, Cycling Stabilities, Areal Capacity Loadings, and Electrode Compositions^a^

a Both p-type and n-type molecules from the class of polymers, small molecules, and covalent and metal–organic frameworks (COFs and MOFs, respectively). Note that organic negative electrodes (anodes)^57−62^ and organosulfur materials^57,63^ are not included in the table. Practical capacities are directly taken from the references and can contain considerable but highly variable amounts of capacitance contributions from conductive carbons used in electrode formulation (as one can see from the last column). The gravimetric energy density is calculated only per cathode active materials by multiplying the potential of the organic electrode vs Li^+^/Li with practical capacity. The results for compounds **14**, **19**, **20**, and **32** are reported in Na cells, but the energy density of the cathode material is recalculated vs Li^+^/Li. The reported electrochemical characterization is exclusively done in Li/Na metal half-cell setups with an excess of a Li/Na electrolyte and metal anodes. Binder and carbon black abbreviations: PVDF, polyvinylidene fluoride; CMC, carboxymethyl cellulose; PTFE, poly(tetrafluoroethylene fluoride); PVA, polyvinylalcohol; VGCF, vapor-grown carbon fiber; CF, carbon fiber; MWCNTs, multiwalled carbon nanotubes; CNTs, carbon nanotubes.^33,35,36,37,38,40,41,42,43,44,45,46,48,49,50,51,53,54,56^

In the past, high-capacity materials were generally limited to conjugated carbonyl materials incorporating several electroactive groups on a small conjugated framework. However, it was shown that small electroactive groups typically suffer from poor stability^16^ and incomplete capacity utilization.^5^ Recently, a new type of compound has been developed by incorporating different functional groups, allowing us to move beyond the theoretical capacity limitations of a single redox moiety. This strategy combines both quinone and phenazine redox functionalities on a hexaazatrinaphthalene (HATN) framework^17,18^ or ladder-type polymers.^19^ High-voltage organic materials were in the past limited to p-type radical organic materials based on the nitroxyl group. Its material capacity is limited because it typically exchanges only one electron per functional group. One of the most commonly applied radical polymers, 2,2,6,6-tetramethylpiperidinyloxymethacrylate (TEMPO-PMMA),^7^ exhibits a redox potential of 3.5 V vs Li^+^/Li and a theoretical specific capacity of 111 mAh g^–1^, leading to a moderate energy density of 390 Wh kg^–1^ on the level of the active material. More recent approaches to increasing the redox potential of organic materials include structural modulation^20^ or the application of new classes of compounds like conjugated sulfonamides.^21^ New n-type organic compounds possess a redox potential beyond 3 V vs Li^+^/Li, which enables the air stability of lithiated active materials, potentially enabling such cathodes to act as a drop-in replacement for current inorganic LIB cathodes. Metal-ion-containing active materials could also open a path toward an anode-free battery cell design, which would greatly simplify the production of next-generation batteries.^22−25^ However, all of these approaches, proven on model compounds, have yet to be expanded to high-capacity organic compounds. Very interesting approaches are also COFs and MOFs (covalent and metal–organic frameworks, respectively), which incorporate electroactive groups into a stable porous framework, potentially enabling good cycling stability and ionic accessibility due to the large surface area.^26−32^ We highlight that the development of organic cathode materials should be directed with a specific application in mind because not all types of organic compounds can satisfy all requirements (e.g., high-energy density, mechanical flexibility, biodegradability, bioderived feedstock, etc.).

An important downside of organic materials is the electronic conductivity, which ranges from 10^–14^ to 10^–3^ S cm^–1^,^64,65^ leading to the need for a larger amount of conductive carbon in the electrode formulation or material structure characterization with conductive support. Only a few organic materials are considered semiconductors, mostly conducting polymers, such as polyaniline, polypyrrole, and polythiophene, belonging to p-type materials with very limited specific capacities.^6,11,66^ The use of different binders and electrode processing conditions can also lead to differences in the electronic and ionic conductivity of organic electrodes. For example, the most frequently used binders, PVDF and PTFE, have completely different properties. While PVDF coats the electrode particles, the use of PTFE leads to the formation of fibril-like networks. Electrochemical performances of organic electrodes with different binders are seldom compared in the literature,^67,68^ but no broad systematic comparison of binders and their effect on electrochemical performance has been performed so far. Nevertheless, the frequent use of PTFE binders in an aqueous suspension demonstrates a clear possibility for the aqueous processing of organic electrodes and the use of aqueous-based binders, as long as active materials are compatible with water processing.

The volumetric density of organic materials is typically ≪2 g cm^–3^, which is 2–3 times lower than that of inorganic cathode materials used in LIBs. The lower density and large amounts of added electron conductive additives have an impact on the practical volumetric energy density and represent important parameters during the electrode engineering process. Unfortunately, those parameters have still not been extensively tested in practice due to the lack of work on organic battery prototyping. Prototyping studies should provide missing insight into the required electrode porosity, tortuosity, and optimal areal capacity, which, together with separator thickness, determine the amount of added electrolyte. Hence, a realistic direct comparison with practical LIBs electrodes is not yet possible. However, conducting such a comparison would be highly beneficial for the identification of future research directions. On the contrary, it has to be noted that due to the high variability of the organic materials, such results might not be fully extrapolated across the whole landscape of organic materials. Several attempts of high areal loadings and decreased carbon contents have been reported in the literature and have demonstrated a certain level of success.^69−72^ To achieve an energy density comparable to that in commercial LIBs, where areal capacities exceed 5 mAh cm^–2^,^73^ very large active material loadings and small amounts of the electrolyte should be targeted. As one can see from Table 1, loadings in laboratory tests are typically well below such numbers and rarely reach 1 mAh cm^–2^.

## Accurate Capacity Determination of Organic Materials

**Figure 2:**
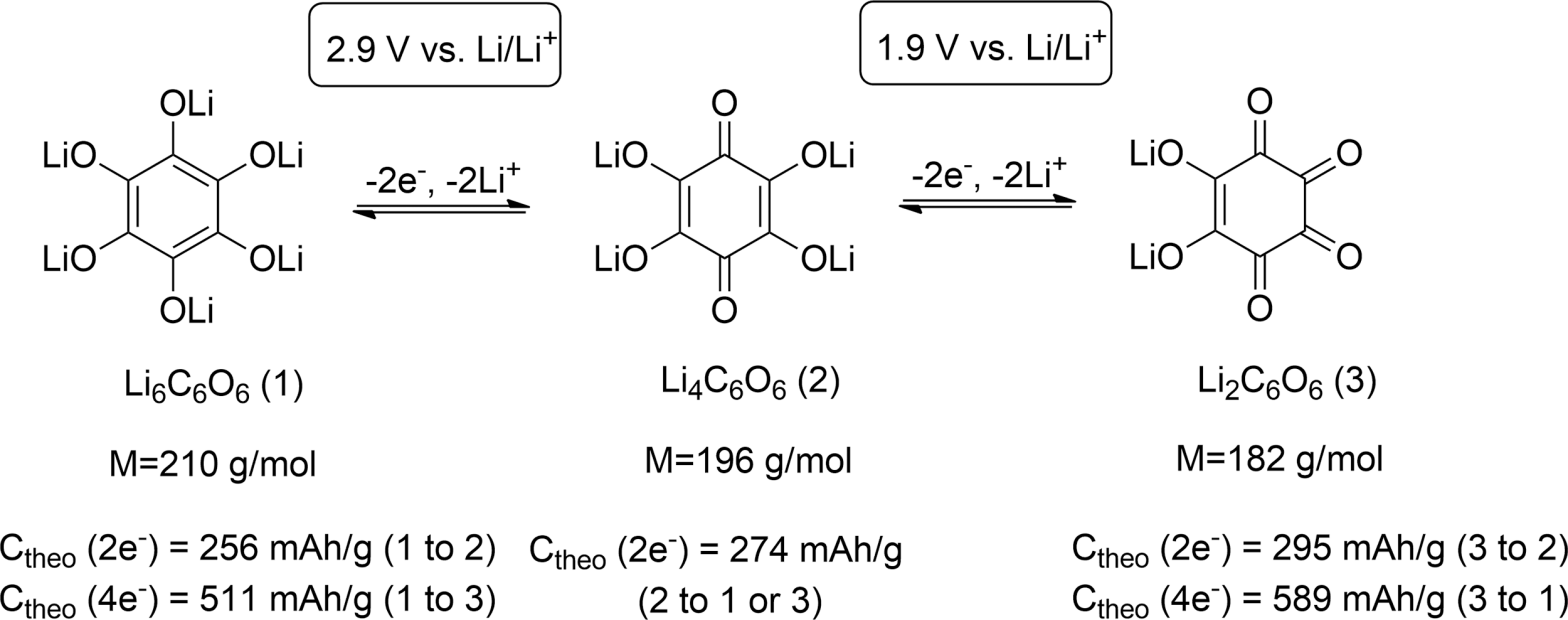
Schematic representation of redox reactions between different redox states of dilithium rhodizonate (Li_2_C_6_O_6_) with their molecular weights and theoretical capacities based on the molecular weight and the number of exchanged electrons.

Turning our focus to specific research on organic electrode materials, we find it is very important to start with the proposed redox mechanism showing oxidized and reduced chemical formulas and the expected number of exchanged electrons (*z*). A proposed and clearly defined electrochemical mechanism with a defined molar mass (*M*) of the active material is the basis for the calculation of the theoretical capacity (*C*_theo_) of the material (eq 1), which is necessary for evaluating the specific capacity. While some redox reactions like benzoquinone reduction are self-evident and easy to understand, the mechanism of more complex organic structures can quickly become too complex for nonspecialists to grasp and derive corresponding theoretical capacities.
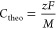
1Figure 2 shows an example of dilithium rhodizonate (Li_2_C_6_O_6_), a well-known organic electrode compound. Li_2_C_6_O_6_ can be reduced to Li_4_C_6_O_6_ at 2.9 V vs Li^+^/Li.^16,74,75^ At a low potential of 1.9 V vs Li^+^/Li, we can reduce it further to Li_6_C_6_O_6_. Depending on the starting material and exchanged number of electrons in the final states (*z*), we can calculate different theoretical capacities ranging from 256 to 589 mAh/g. Hence, the initial state of the active material and the corresponding theoretical capacity with the proposed electrochemical reaction should always be clearly stated.

Evaluation of the obtained specific capacity is a part in which analysis in the literature is often not performed with sufficient rigor and can lead to misleading conclusions and poor reproducibility among different laboratories. Thus, we propose a more rigorous electrochemical testing procedure for calculating the specific capacity of compounds and the subtraction of the contribution of conductive additives. As mentioned before, the amounts of conductive additives (typically CB) in the organic electrodes are much larger than in the case of electrodes comprised of inorganic active materials. Typically, relatively large amounts of CBs are used to have a one-size-fits-all recipe for electrode preparation, which ensures the good performance of organic compounds with variable conductivity and particle size, which does not require compound-specific optimization of electrode preparation. A typical electrode preparation recipe is 60 wt % active material, 30 wt % CB, and 10 wt % PTFE or PVDF binder. Surprisingly, 30 wt % CB is quite a moderate value in the literature (where amounts exceeding 50 wt % can often be encountered).^47,76−83^ Nevertheless, it is still a very large amount leading to a significant contribution of CB with capacitance behavior. Furthermore, a significant number of studies utilize large-surface area CBs (Ketjen Black, Printex XE2) and wide electrochemical windows. Another issue of large-surface area CBs is the increased surface area for potential side reactions, which might become highly detrimental in practical cells and lean electrolyte conditions and are not evaluated in typical laboratory cell setups.

To obtain an actual capacity of the organic electrode material, the CB contribution needs to be subtracted from the measured capacity (*Q*_AM+CB_), which typically consists of two contributions: capacity of organic active material *Q*_AM_ and capacitance of CB *Q*_CB_.
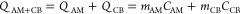
2where *Q*_AM_ can be expressed as a product of the mass of active organic material *m*_AM_ and specific capacity *C*_AM_ of the material, and the same is valid for *Q*_CB_. In the literature, the measured capacity of the whole electrode normalized by the mass of active material (*Q*_AM+CB_/*m*_AM_) is most often listed as the “specific capacity of active material” without stating the contribution to this value due to the capacity of the carbon black (*C*_CB_). We will call *Q*_AM+CB_/*m*_AM_ measured specific capacity *C*_meas_. By rearranging the eq 3, we can express the real specific capacity of the active material.
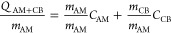
3
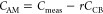
4To obtain the real specific capacity of the active material, we need to subtract the specific capacity of carbon black *C*_CB_ normalized with the ratio (*r*) between the mass of carbon black and the active material in the electrode (eq 4). With the formulation mentioned above, 60 wt % active material and 30 wt % carbon black, the normalization factor is 0.5. The specific capacity of Printex in the given voltage window was measured to be 33 mAh/g,^84^ which means that we should subtract 16.5 mAh/g from the measured capacity to obtain the true specific capacity. Although, in this example, the difference is not huge, it can be much more significant if the content of CB is higher due to the increase in the normalization factor, which can, in extreme cases, even reach 8 (e.g., 80% of CB and only 10% of active material, which is evident from Table 1).^34,39^ It is important to note that the capacity of CB should be measured at different current densities to account for the potential redox activity at different current densities, as well as potential pseudocapacitance and the contribution of side reactions at very low current densities. All capacitive contributions should be removed if additional inactive material is present, e.g., carbon nanotubes or graphene-type materials used to nanostructure the active material.^10,84^ Measurement of the CB capacity contribution (*C*_CB_) can be done by electrochemical testing of the electrode composed of only CB and binder under the same conditions that were used for active material testing. Such a test has the additional benefit of verifying the operating voltage window for electrochemical characterization. Electrolyte stability windows (ESW) are typically evaluated on the small-surface area metal electrodes with relatively high sweep rates by the linear sweep or cyclic voltammetry, which might lead to unrealistically wide ESWs. Thus, the ESW should be verified with electrodes exhibiting a surface area similar to that of the active material containing electrodes.

**Figure 3:**
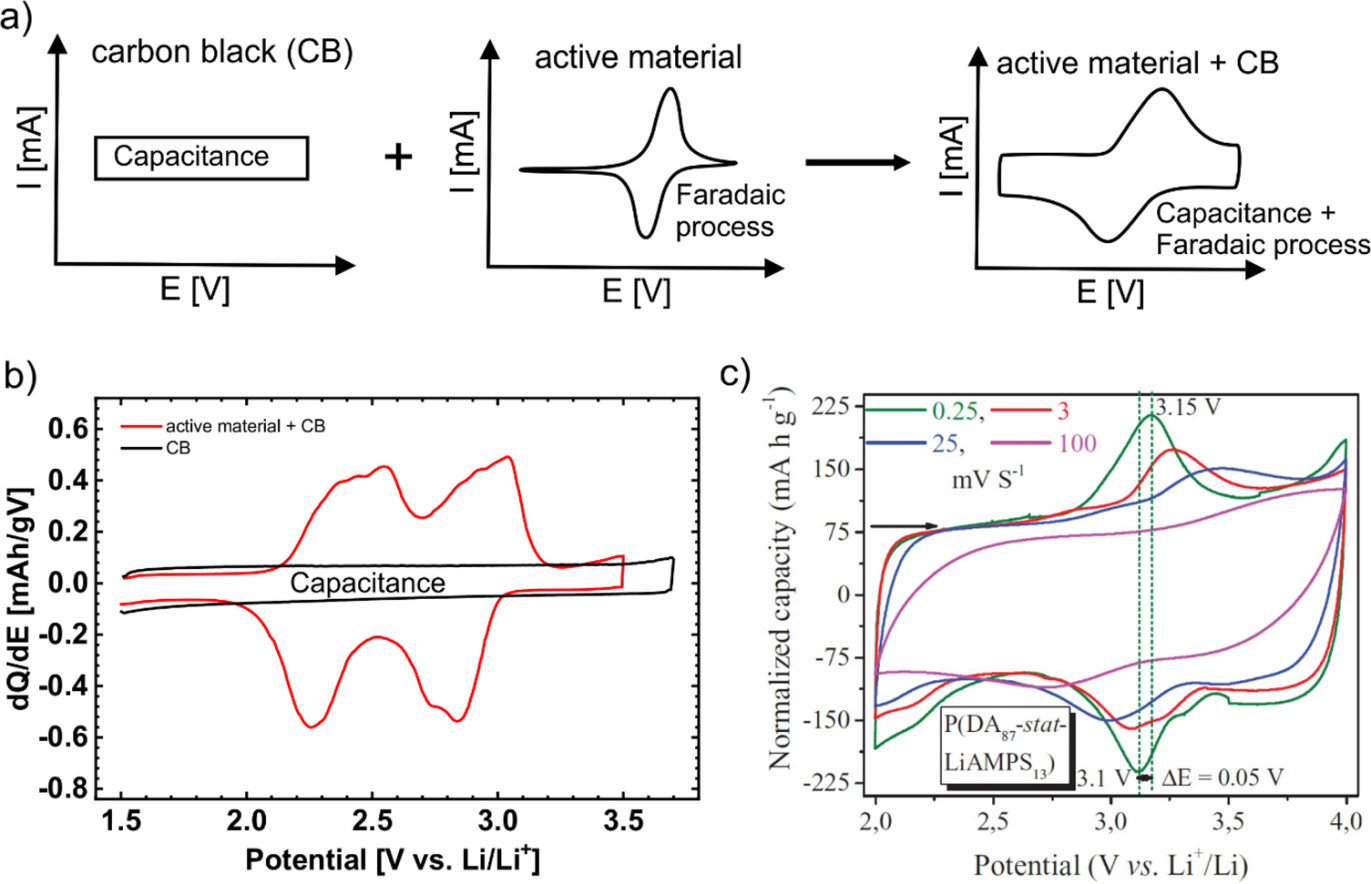
(a) Schematic presentation of CV responses of carbon black (CB) and organic active material and summary of their electrochemical response in the electrode, which is what we typically obtain during characterization of organic electrodes. (b) Polyphenanthrenequinone with 42.6% carbon additive (red) and carbon additive blank electrode (black) used in the formulation as a comparison.^84^ (c) Catechol-based copolymer with 80% carbon additive [carbon nanotubes (CNTs)].^47^ Panel (c) is reproduced with permission from ref (47). Copyright 2014 John Wiley and Sons.

Qualitatively, the contribution of the capacity from CB can be visualized from the shape of the cyclic voltammogram (CV) or d*Q*/d*E* derivative of galvanostatic cycling. CB acts like a capacitor, and in the ideal case, the shape of the CV is rectangular without discernible redox peaks. The plain active material, on the contrary, typically provides a distinctive shape with more or less defined redox peaks. However, certain active materials exhibit a capacitance-like response due to their large surface area or specific redox mechanism. In practical measurements, we typically obtain a combination of active material and CB contributions because electrodes always contain a certain amount of CB (Figure 3a). Comparable curves are also obtained when galvanostatic measurements are converted to d*Q*/d*E*. The first example is poly(phenanthrene quinone) with 42.6% carbon additive (Printex CBs and graphene-type material used for nanostructuring of the active material)^84^ (Figure 3b), and the second is a catechol-based copolymer with 80% carbon additive [carbon nanotubes (CNTs)] (Figure 3c).^47^ In the latter example, very small redox peaks and a large capacitance can be observed, leading to the potential conclusion that a very significant share of the measured capacity of the electrode comes from CNTs and not from the active material. Most often, the large amount of added CBs consequently leads to low areal loadings and enables electrochemical tests at a relatively high current density, targeting the high gravimetric power of organic materials. The high-power performance typically exposes the advantages of organic materials and is often used in the literature as a clear advantage of organic materials over inorganic ones. However, we encourage researchers to perform complementary tests at lower current densities to evaluate the stability of organic materials, the presence of side reactions, and shuttling phenomena more thoroughly,^85^ as they might be key parameters for real-life application. Together with the estimation of the CB contribution, such analysis would go a long way toward improving the comparability between different organic materials tested in variable laboratory setups by different research groups, as well as practical application. We strongly believe that conducting control experiments is crucial for every investigation involving CB in the electrode formulation. Therefore, we propose that including control experiments as a standard requirement in the Supporting Information should be adopted in all future publications.

## Evaluation of the Obtained Electrochemical Capacity

The specific capacity (eq 4) can be either much lower than the theoretical, close to theoretical, or higher than the theoretical capacity. In the sections below, we will try to explain the most likely reasoning behind each of these three scenarios.

**Figure 4:**
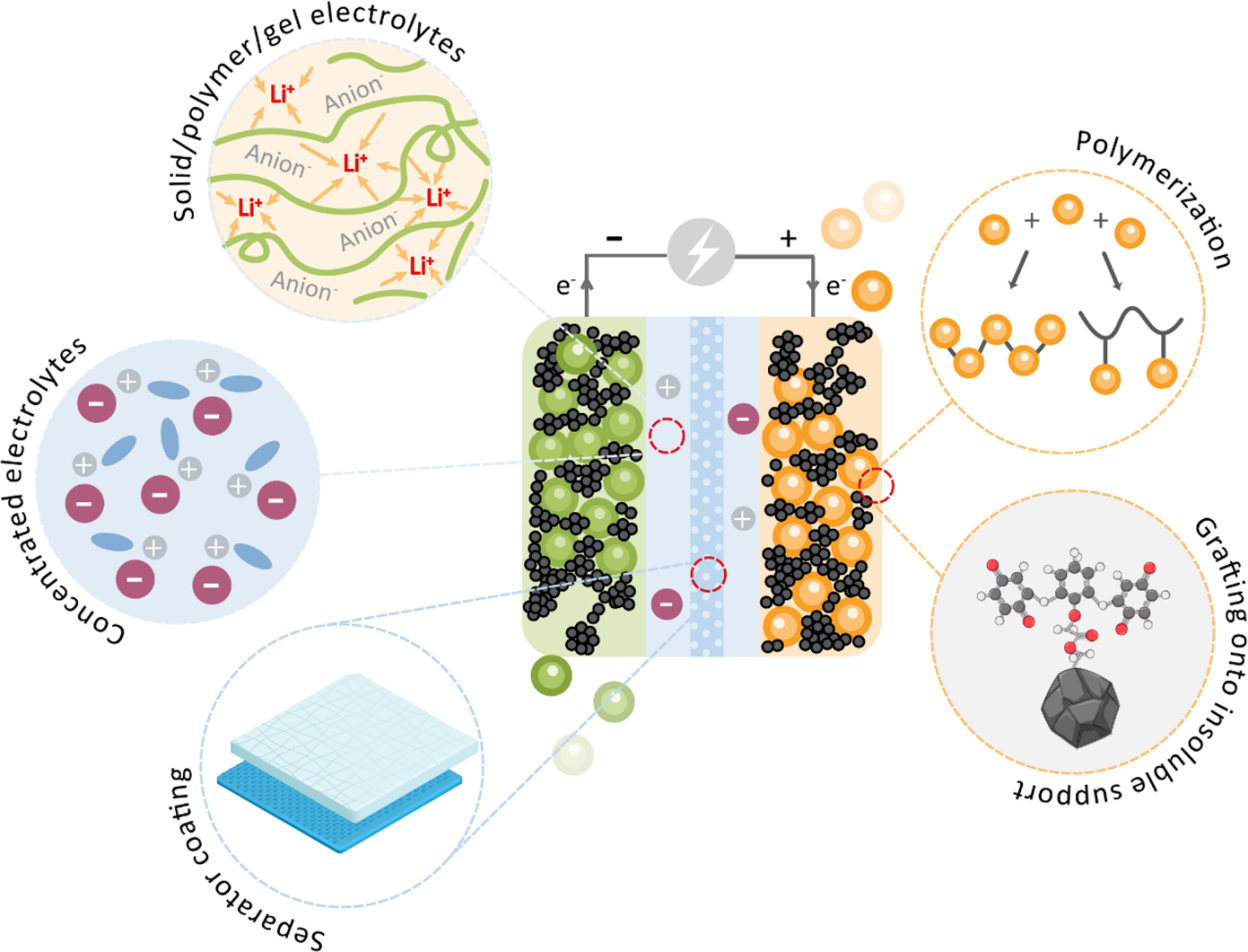
Different strategies that can be employed for the mitigation of capacity fading due to the dissolution of the active material.

The obtained capacity can be much lower than theoretical due to (i) a low level of utilization of the active material, (ii) a fast capacity drop, or (iii) poor electrode engineering. Typically, the reason behind the low level of utilization of the active material is the poor electrolyte accessibility to electroactive groups due to limited swelling.^86^ This often occurs in the case of rigid, cross-linked materials exemplifying poor electronic and ionic conductivities. The swelling of organic compounds is electrolyte-dependent and can be improved by changing the solvents.^67^ A fast capacity drop most often occurs due to the high solubility of the active material, which can be, in certain cases, accompanied by shuttling of redox active species, resulting in a very low Coulombic efficiency.^85^ Solubility issues can be addressed by electrolyte tuning targeting the lower solubility of active materials in an electrolyte or by increasing the concentration of the salts or variation of solvents.^87−89^ Other methods for limiting the dissolution of active material are being grafted onto an insoluble support,^90,91^ infiltration inside mesoporous materials,^92−94^ use of ionoselective membranes,^95^ use of ionic liquids,^89,96^ semipermeable electrolytes (ceramic, polymer, and gel),^89,97−100^ and polymerization (Figure 4).^57,58,60,63^ However, certain approaches only slow or delay dissolution, and special care has to be taken to evaluate dissolution during prolonged cycling at low rates. An important consideration is also to prevent dissolution, not only in a pristine state of the active material but also in other redox states. According to the number of publications and literature trends, the most widely used method is polymerization. Although it requires additional synthesis and adds the molecular weight of the linker to the electroactive group, other options typically lead to a more significant decrease in the energy density and/or make battery assembly much more complex.

In certain cases, the achieved capacity is close to the theoretical one, which could indicate that the proposed redox reaction is correct and the bulk of the material is successfully utilized in the electrochemical reaction. However, it can also be caused by serendipity and the interplay of different electrochemical reactions. Hence, direct proof should be obtained through the estimation of the percentage of the reacted active material and confirmation of the proposed redox reactions, which will be discussed in the following section. The measured capacity can be larger than the theoretical value for several reasons. Most frequently, the measurements are performed beyond the electrolyte stability window (ESW). This happens quite often, due to the improper evaluation of the ESW, as discussed above. Thus, the stability window of the electrolyte should be validated in an environment as similar as possible to that of practical cell tests. The simplest approach is to measure the stability window of the testing electrolyte with “control electrodes” and afterward proceed with the measurement of an “organic electrode” composed of the active material, CB, and the binder. The ESW is strongly dependent on used solvents and salts, with ether-based electrolytes displaying lower oxidative stability than carbonate electrolytes. Without ESW verification, the side reactions can be easily misinterpreted as the electrochemical response of the active material, especially when operating in a very wide electrochemical window and excess electrolyte conditions, leading to the overestimation of material activity. Electrochemical characterization is an essential, simple, and cost-effective tool for battery characterization. However, its interpretation can be highly biased, and the performance of organic materials is often overestimated by assigning electrode electrochemical activity exclusively to active material. Hence, we propose a more rigorous approach, an electrochemical characterization that is always supported by the understanding of the mechanism and control experiments for potential side reactions. Such an approach should improve the comparability between different materials and laboratories and allow more realistic benchmarking of organic electrodes versus LIB standards, leading to the accelerated development of organic materials.

## Investigation of the Electrochemical Mechanism

Investigation of the organic electrode mechanism is a key point for the validation of the proposed electrochemical mechanism needed for the evaluation of the practical capacity. The variability of the organic compound landscape, as well as the poor crystallinity of most organic compounds, has led to the fact that their electrochemical mechanism has been investigated in less detail. However, their electrochemical response is usually a combination of diffusion-controlled faradaic reaction storage in bulk and surface-controlled faradaic reactions at or near the surface of the material (pseudocapacitance) and can be distinguished by running CV tests at various rates.^101^ The specific ratio between the two electrochemical responses is influenced by different parameters like the active material type, particle size, binder, carbon additives, electrolyte amount, electrode porosity, and thickness. Researchers in the past have mainly focused on the electrochemical performance and less on the mechanism due to a lack of accessible characterization tools. Today, with the wide accessibility of advanced characterization tools, we can analyze electrochemical mechanisms through the application of complementary analysis techniques by using organic electrodes and/or model compounds. On the other hand, experimental work can be complemented by computational modeling. Computational modeling enables complementary insights into redox potentials, the number of exchanged electrons, the thermodynamic stability of electrode compounds, and their vibrational analysis.^102^ While many properties can be modeled using more straightforward high-precision density functional theory modeling tools, more complex and amorphous organic materials, such as polymers, require the use of molecular dynamics (MD) modeling, which allows the study of much larger systems on a longer time scale. A lack of focus on mechanistic investigation is evident from the literature reviews, which in most cases focus on extensive comparison of the electrochemical performance of organic materials but often fail to provide a critical overview of analytical techniques that can be applied to study the electrochemical mechanism of organic materials.

**Figure 5:**
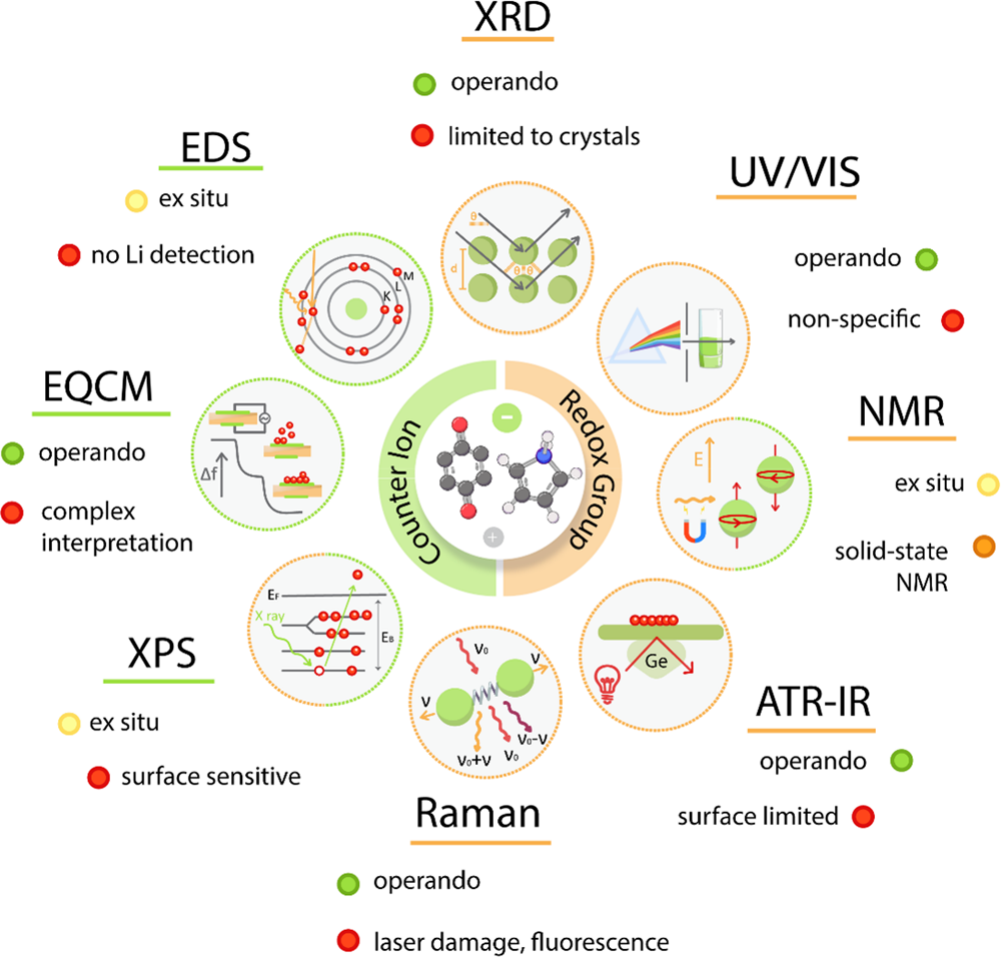
Overview of different characterization techniques for investigation of the electrochemical mechanism of organic compounds with their modes of operation and typical limitations. If a specific method enables *operando* characterization, *in situ* characterization and *ex situ* characterization are possible.

The electrochemical mechanism can be investigated through *ex situ*, *in situ*, and *operando* methods, all of them having their strengths and weaknesses. While *ex situ* characterizations are the simplest and most widely applied, they often carry uncertainty regarding sample degradation during the disassembly of the cell, washing of electrodes, and sample handling.^103−105^ Application of *in situ* and *operando* techniques removes the concerns regarding the sample degradation and allows detection of intermediate states, but it is often difficult to perform because analysis signals from the relatively bulky battery cell setup and thick electrodes can be difficult to extract. This can be mitigated by the use of dedicated cells or high-intensity probes, which can sometimes be achieved only through the use of synchrotron radiation. On the contrary, upon application of *in situ* and *operando* modified battery cell setups, special care needs to be taken to validate the transferability between the conventional battery cells and characterization-dedicated cells due to modified cells leading to the different amounts of electrolyte, different areal capacities, changes in the geometry, and, in case of high-intensity probes, sample damage. The most used laboratory battery cells are coin cell and pouch cell formats. Both can be adapted to incorporate different probe windows, which can enable infrared (IR), Raman, ultraviolet–visible (UV–vis), X-ray absorption near edge structure, X-ray Raman spectroscopy (XRS), and other characterization techniques. Although the current literature typically relies on *ex situ* results, more effort should be invested into complementary and supportive experiments investigating the effect of washing, disassembly, passive layer formation, and potential sample degradation during battery disassembly and post-treatment. In the following section, we will briefly take a look at specific techniques, their strengths, and their limitations (Figure 5).

X-ray diffraction (XRD) is one of the most general and explored tools in the study of inorganic cathodes and can be quite easily applied *operando* or *in situ*. Together with structural analysis, it can be a very powerful tool for the analysis of crystalline compounds.^106−108^ XRD is typically limited to small crystalline compounds and cannot be applied to organic materials in general, especially polymers, which are typically amorphous. IR spectroscopy is another widely applied characterization tool for the analysis of organic materials. It can be performed in different setups in transmittance or attenuated total reflectance (ATR) mode. Transmittance can give us insight into the bulk of the electrodes but is limited to *ex situ* samples because it requires sample dilution and preparation of the pressed pellets in an inert atmosphere. On the contrary, ATR enables direct measurements of the electrodes but suffers from surface sensitivity, which means that we usually probe only a few outer micrometers of the electrode (typical thickness in the range of 100 μm), which causes overrepresentation of surface phenomena and leads to a lack of information from the bulk. Surface analysis is especially problematic when using soluble organic materials and/or having an inhomogeneous electrochemical response along the thickness of the electrode.^109^ A limitation of IR characterization is that many IR bands fall into the fingerprint region and overlap with signals from the electrolyte, binder, and other cell components. Thus, assignment and interpretation can be complicated, and extensive comparison of the electrode measurements with synthesized model compounds, literature references, and theoretical calculations should be pursued.^103,110^*Operando* ATR-IR enables continuous measurement and identification of intermediate states that might not be self-evident from the shape of galvanostatic curves.^109^ However, it typically requires a subtraction procedure to remove bands of inactive cell parts. Raman spectroscopy is often considered to be complementary to IR spectroscopy and can be performed with broadly available spectrometers. Nevertheless, it is often plagued by laser damage or sample fluorescence, limiting its practical applicability.^111−113^

An interesting technique that can be applied to conjugated organic compounds is UV–vis spectroscopy, but interpretation without suitable model compounds or theoretical calculations can be limited due to nonspecific changes.^76,114^ X-ray photoelectron spectroscopy (XPS) is a surface-sensitive technique that allows us to probe the chemical nature of organic compounds and even provide the elemental composition. However, XPS is even more surface-sensitive than ATR-IR, as its probing depth is typically in the range of several nanometers. Therefore, extra precautions should be taken to prevent sample degradation during disassembly and washing.^113,115−119^ Interpretation of XPS is strongly influenced by the peak fitting process, and quantification is possible only through systematic investigation of standard samples, which is unfortunately rarely done in the battery literature.^120^ XPS investigation within the bulk of the electrodes can be performed with the help of sample sputtering, but sample degradation during the sputtering should be carefully investigated beforehand. Nuclear magnetic resonance (NMR) is another common analysis technique for organic compounds,^121−123^ but because most organic electrodes contain insoluble active materials, solid-state NMR is needed. Solid-state NMR typically requires a larger amount of material and is limited to *ex situ* samples.^28,55,104,108,124,125^ A relatively similar yet even more sensitive technique is electron paramagnetic resonance (EPR), which is limited to the study of radical species.^106,126^ X-ray absorption spectroscopy (XAS) for organic materials is relatively difficult to use due to the low energy of the K edge of light elements (C, O, and N), which puts them into the soft X-ray range and limits the penetration depth and analysis to the surface of the sample.^127^ This can be avoided by using inelastic scattering of hard X-rays on shell electrons, so-called XRS. XRS is a bulk analysis technique that can be used semiquantitatively to monitor the level of electrochemical conversion and has only recently found application in organic materials.^128^ Although electron microscopy is extensively used to study the mechanism and degradation of inorganic active materials, its use in the field of organic electrode materials is somehow limited to qualitative assessments of electrode morphology, dissolution phenomena, and detection of passive layers.^52,129,130^

As described above, there are a plethora of analysis techniques devoted to the determination of the reaction mechanism in organic electrode materials. However, only limited emphasis was put on the nature of the metal cations involved in the electrochemical process. Most often, studies predict an exclusive cation coordination mechanism (e.g., Li^+^ or Mg^2+^). However, ion pairs were detected in multivalent electrolytes (e.g., MgCl^+^). Currently, the most frequently applied analytical techniques for monitoring the nature of cations are energy-dispersive X-ray spectroscopy (EDX) coupled with electron microscopy^127^ and XPS,^113^ which are both surface-limited (EDX typically to the micrometer range and XPS to the nanometer range) and affected with potential passivation layer formation on the interface between the active material and electrolyte. These two characterization techniques can identify the presence of anions, which contain heterogeneous atoms (S, F, Cl, and P). However, the detection of solvent molecules is much more difficult because they often contain only C and O atoms. Solvent molecules can be detected through IR spectroscopy^76^ or NMR, but special care should be taken to carefully dry the samples to remove residues of electrolyte solvents and solvents used for washing.^76^ A very powerful yet somehow underutilized technique is electrochemical quartz crystal microbalance (EQCM), which allows tracking of the gravimetric changes of the electrode during electrochemical cycling. Its practical application for porous electrodes is not straightforward because electrodes in contact with electrolytes are subjected to various phenomena connected with changes in their mechanical properties and the viscoelastic properties of the electrolyte, which make direct gravimetric observation difficult.^131^ So far, EQCM characterization has been mostly limited to aqueous solutions, where it has revealed different amounts of water being co-inserted with different types of mono- and bivalent cations.^132^

The underutilized approach in mechanistic investigation is the complementary chemical synthesis of different redox phases instead of only electrode analysis. Direct synthesis allows significantly simpler characterization in bulk samples and unambiguous assignment of signals.^133^ On the contrary, electrode characterization might lead to a poor signal due to the presence of other electrode components, passive layers, and electrolyte residues. Direct chemical synthesis of different redox states might be challenging for organic materials with limited processability like polymers but can always be performed on model-type compounds.^103,110^

## Post-Li Metal–Organic Batteries

In the last part, we will take a look at post-Li metal–organic batteries, where the application of organic electrodes has been popularized due to the severely limited performance of inorganic cathodes in certain systems. The most direct alternative to Li batteries is to consider metal–organic batteries that utilize Na and K as metal anodes. When using Na- and K-based electrolytes, organic electrodes demonstrate favorable reversibility; however, there may be a reduction in both capacity retention and utilization.^15,134^ The diminished electrochemical performance can be attributed to interactions with various cations, as well as the more limited selection of electrolytes. Unfortunately, the low melting points of Na and K metals present a serious safety issue that might prevent their commercialization.

**Figure 6:**
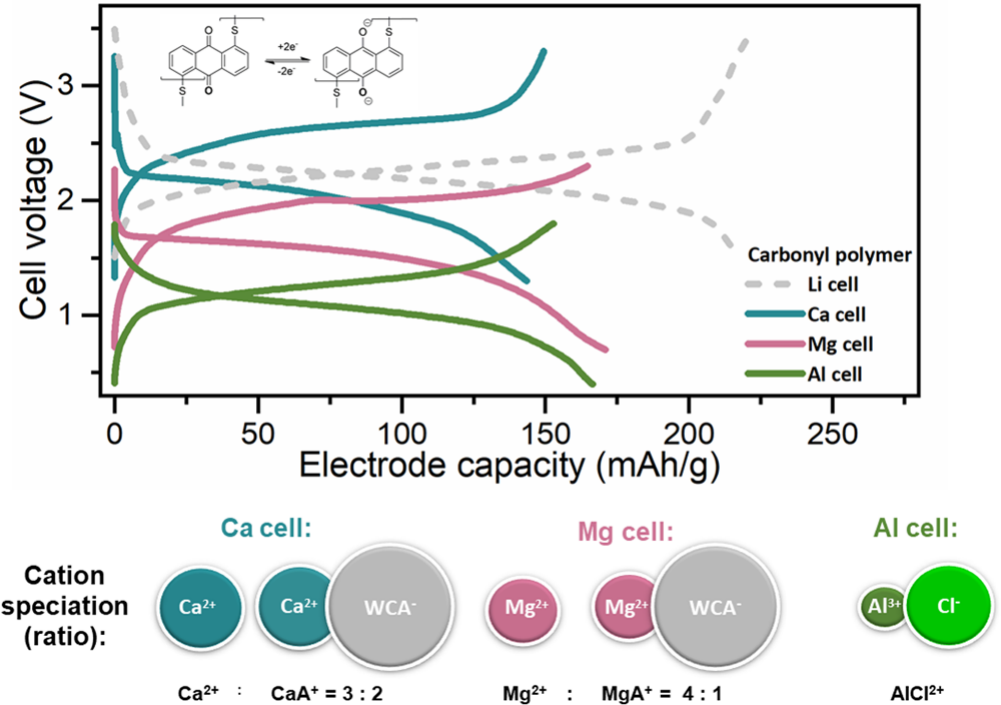
Voltage and capacity comparison of an anthraquinone-based polymer cathode in different multivalent metal (Ca, Mg, and Al, Li as a benchmark) anode cells. Cationic speciation is estimated from the EDX analysis of *ex situ* electrodes. Data are replotted from previous publications of our group.^105,118,149^ Mg and Ca electrolytes were based on WCA-fluorinated alkoxy borate salts, while the Al electrolyte was based on AlCl_3_ salt.

Multivalent (Mg, Ca, and Al) metal anodes offer high gravimetric and volumetric capacities that surpass the gravimetric capacity of the graphite anode of standard LIBs.^135^ While Zn metal does not offer gravimetric capacities as high as those of Mg, Ca, and Al, it enables the use of aqueous electrolytes and simplified manufacturing due to the stability in the ambient atmosphere. Hence, research on Zn metal–organic batteries has been recently popularized and is a plausible possibility for stationary storage.^136−141^ From the viewpoint of high energy density, a combination of multivalent metal anodes with a low redox potential and high-energy organic cathodes is especially interesting; e.g., the combination of 1,4-benzoquinone and a Mg metal anode offers a high theoretical energy density of 810 Wh kg^–1^ on the level of the electrode materials.^142^ Unfortunately, the development of multivalent organic batteries is still plagued by the limited number of practically applicable electrolytes. One of the reasons is that organic materials typically contain electrophilic groups, which prevent the use of nucleophilic electrolytes such as Grignard reagent or BH_4_^–^-based electrolytes. In recent years, non-nucleophilic Mg and Ca electrolytes were developed^143,144^ and enabled broader applications of organic cathodes.^4,105,145,146^ Although organic materials undergo an equivalent electrochemical mechanism and offer good electrochemical reversibility by far surpassing inorganic cathodes in multivalent electrolytes (Figure 6),^109,128^ their capacities and long-term stability quite often fall short of the practical capacities achieved in Li counterparts.^147^ Lower practical capacity utilization is an interplay of worse electrolyte accessibility to the organic active groups and the higher overpotentials of both the anode and the cathode. Alkali metal plating/stripping overpotentials are typically very small and lead to negligible or minor overpotential contributions in the cycling of metal–organic two-electrode half-cells. On the contrary, multivalent metal anodes typically have much larger overpotentials.^148^

As mentioned before, the coordination mechanism with ion pairs (e.g., MgCl^+^) can be a dominant charge-storage mechanism.^109,147^ Namely, the inclusion of cation–anion pairs in the electrochemical mechanism greatly increases the amount of electrolyte salt needed for the reversible operation of the electrochemical cell and severely limits the practical energy density.^104^ This can be alleviated by the use of chloride-free Mg salts with weakly coordinating anions (WCAs). However, even in the case of a WCA based on a fluorinated alkoxyborate anion, a sizable contribution of cation–anion pairs was detected in discharged cathodes (Figure 6).^105,149^ Therefore, future research on multivalent electrolytes should focus on designing electrolytes that exhibit facile ion dissociation. In the case of Al batteries, electrolytes enabling reversible Al metal plating/stripping are based on AlCl_3_, and it is still not clear which ionic species (AlCl^2+^ or AlCl_2_^+^) are prevalent in the n-type cathode electrochemical mechanism.^104,146,150,151^ To move multivalent batteries toward high-energy applications, there is a clear need for the improved dissociation of multivalent cations inside electrolytes to be able to minimize the amount of the electrolyte and enable operation under lean electrolyte conditions. In general, the development of multivalent metal–organic batteries needs to progress in several key areas, particularly in terms of multivalent cation dissociation, practical capacity utilization, and long-term cyclability. While the two latter issues could be mitigated by the synthesis of organic compounds with improved electrolyte accessibility and stability, improved dissociation of multivalent cations will require significant work on the use of different solvents and salts or the use of additives that could manipulate the cation solvation structure.^152^

## Conclusions

Organic electrode materials as sustainable and low carbon footprint materials have great potential for future battery technologies. However, most of the practical development of organic batteries is still on the level of technology validated in laboratory half-cells. More efforts should be focused on the most promising materials for practical application to evaluate their performance in prototype cells and identify potential shortcomings, which should be addressed in future research. At the same time, the literature has provided many exciting results in recent years, in the areas of both new high-voltage and high-capacity organic compounds that need to be developed further to ensure long cycle life. However, many of the literature reports have employed testing conditions that might have underestimated the potential shortcomings of organic compounds, such as high rate cycling, low areal loadings, and high electrolyte/active mass ratios. In the vast majority of studies, the specific capacity that is related to the material also includes the electrochemical activity of additives. Altogether, this makes a head-to-head comparison of organic materials very difficult and might present unexpected challenges when moving toward practical application. The latter requires a high areal loading of electrodes, achieving high cycling efficiency, and decreasing the CB content and the amount of the electrolyte. More effort should be dedicated to understanding the organic mechanism, where characterization should be done with several complementary techniques and supported by analysis of model compounds and computational modeling to ensure the most precise interpretation.

Nevertheless, organic materials outperform inorganic materials by far in post-Li battery technologies and have shown tremendous progress in recent years. This is especially true in the field of multivalent batteries, where they currently offer the most realistic possibility for commercialization. Hence, we strongly believe that with increased rigor put into electrochemical testing and material characterization, researchers should be able to better “separate the wheat from the chaff” and enable organic material-based batteries as a realistic future alternative, not just a distant mirage.
